# Mining non-model genomic libraries for microsatellites: BAC versus EST libraries and the generation of allelic richness

**DOI:** 10.1186/1471-2164-11-428

**Published:** 2010-07-12

**Authors:** Christopher K Ellison, Kerry L Shaw

**Affiliations:** 1 Department of Neurobiology and Behavior, Cornell University, Ithaca, NY 14850, USA

## Abstract

**Background:**

Simple sequence repeats (SSRs) are tandemly repeated sequence motifs common in genomic nucleotide sequence that often harbor significant variation in repeat number. Frequently used as molecular markers, SSRs are increasingly identified via *in silico *approaches. Two common classes of genomic resources that can be mined are bacterial artificial chromosome (BAC) libraries and expressed sequence tag (EST) libraries.

**Results:**

288 SSR loci were screened in the rapidly radiating Hawaiian swordtail cricket genus *Laupala*. SSRs were more densely distributed and contained longer repeat structures in BAC library-derived sequence than in EST library-derived sequence, although neither repeat density nor length was exceptionally elevated despite the relatively large genome size of *Laupala*. A non-random distribution favoring AT-rich SSRs was observed. Allelic diversity of SSRs was positively correlated with repeat length and was generally higher in AT-rich repeat motifs.

**Conclusion:**

The first large-scale survey of Orthopteran SSR allelic diversity is presented. Selection contributes more strongly to the size and density distributions of SSR loci derived from EST library sequence than from BAC library sequence, although all SSRs likely are subject to similar physical and structural constraints, such as slippage of DNA replication machinery, that may generate increased allelic diversity in AT-rich sequence motifs. Although *in silico *approaches work well for SSR locus identification in both EST and BAC libraries, BAC library sequence and AT-rich repeat motifs are generally superior SSR development resources for most applications.

## Background

Microsatellites, or simple sequence repeats (SSRs), are common features of eukaryotic genomes and can be characterized as generally short, repeated nucleotide sequence elements arrayed in tandem and flanked by non-repetitive regions (reviewed in [1]). SSRs often harbor high levels of polymorphism in terms of repeat number and have been developed into one of the most common classes of genetic markers due to their high degree of reproducibility, ubiquity, codominance, and variability among individuals [2-4]. The multi-allelic nature of SSR loci is thought to derive principally from errors occurring due to slipped-strand mispairing during DNA replication [5-8], however, SSRs may also be generated via alternative means, such as retrotransposition events, interhelical junctions forming during chromosome alignment, unequal crossing over, or gene conversion [9].

Frequency of SSR loci is markedly variable across genomes [10-12], although the broader principles of their genomic organization remain poorly understood [13]. Many features that shape genome evolution generally, such as nucleotide composition, may play a large role in the variability of microsatellite density across the genome (e.g. [14,15]). Generally, length and density of microsatellites increase with genome size [16], but several exceptions to this rule have been observed (e.g. [13,17]). Genomic regions of different functionality often maintain SSR loci with different properties. For example, SSRs located within coding regions tend to have an excess of trinucleotide repeats relative to other repeat classes and a specific excess of (CAG) _n _SSR loci [18]. This is generally attributed (1) to the fact that length variant trinucleotide SSRs maintain the appropriate reading frame within the coding region and (2) to the observation that glutamine (CAG) repeats have fewer detrimental effects within a protein than many other repeated amino acids [19]. Further, the striking variation of abundance in exonic, intronic, and intergenic regions suggests that selection might play a role in the genomic distribution of SSR loci (e.g. [20,21]). The number of allelic length variants associated with an SSR locus typically increases with increasing average repeat number at that locus [22,23] (but see [24]), however, and allelic diversity is thought to be primarily a consequence of physical parameters and structural properties of the SSR sequence motif [15].

Parallel to the rapid increase in availability of diverse DNA sequence data, highly labor-intensive methods for the generation of SSR genomic markers (e.g. [25,26]) have been gradually replaced by *in silico *data mining approaches using genomic sequence databases (e.g. [4,27,28], but see [29]). Two such sources are sequence databases derived from expressed sequence tag (EST) and bacterial artificial chromosome (BAC) libraries. Both types of libraries contain sequenced genomic fragments that are effectively randomly distributed throughout the genome; however, they differ in being comprised of actively transcribed components only in the case of EST libraries versus random genomic fragments in the case of BAC libraries. Although transcribed, EST library derived SSR loci still maintain allelic variability comparable to that in non-transcribed genomic DNA [30] and serve as excellent molecular markers for many applications [31].

A number of genomic tools have been brought to bear in investigations of the rapidly radiating Hawaiian cricket genus *Laupala*, including an EST library with 10.17 Mb nucleotide sequence [32] and a BAC-end sequence library with an additional 1.71 Mb of genomic DNA sequence (unpub. data). *Laupala *is a unique evolutionary model system, with one of the highest documented rates of speciation known among invertebrates [33,34]. Members of the genus are often morphologically and ecologically indistinguishable and species can differ by less than 0.1% nucleotide divergence at nuclear loci [35] but extensive divergence is observed in mate-recognition related behavioral characters [36,37]. Additionally, *Laupala *appears to have reduced rates of DNA loss and maintains a relatively large genome size (approximately 11X that of *Drosophila melanogaster *[38]). Consequently, the *Laupala *genus has more developed genomic resources than most Orthopteran groups, allowing a broader investigation of the structural properties of allelic SSR variation in this important group of insects.

Here, we present a survey of 288 unique SSR loci in two species of *Laupala*, *L. kohalensis *and *L. paranigra*, identified in BAC and EST library sequence databases from *L. kohalensis*. The overall distribution of SSR loci within these genomic libraries is examined in addition to the distribution of allelic richness across all SSR loci to evaluate (1) whether SSR density or mean repeat number is elevated in a species with a relatively large genome size, (2) whether particular SSR motifs are particularly common in library sequence and (3) whether the same motifs are likely to harbor significant allelic variation. We further evaluate the structural properties of the SSR loci associated with elevated numbers of length variants and assess the efficacy of BAC and EST library nucleotide sequences for the development of informative molecular markers.

## Results

### Comparison of repeat structure in BAC and EST genomic libraries

SSRs were generally more abundant and comprised of lengthier repeat structures in BAC library sequence than EST library sequence (Table 1). In total, 186 SSR loci were identified in BAC library sequence (1.71 Mb) and 550 in EST library sequence (10.17 Mb); of these, we were able to design primers in flanking sequence for 135 and 435 loci, respectively. Primers could not be designed for 49 and 108 SSR loci from the BAC and EST libraries, respectively, that were located at a terminal sequence end and for an additional 7 SSR loci due to lack of suitable priming sites. SSRs were approximately two to three times more likely to be identified in a BAC versus an EST library sequence (*X*^2 ^= 144, df = 1, p > 0.0001) and SSRs comprised nearly five-fold higher percentage of all sequenced bases in BAC library sequence (*X*^2 ^= 6.8, df = 1, p = 0.0091). Further, SSRs identified in BAC library sequence were comprised of significantly more repeats than were those in EST library sequence (Mann-Whitney test p < 0.0001) (See Table 1).

**Table 1:** SSR genomic sequence distribution summary statistics

	BAC library	EST library
Reads • SSR^-1^	11.89	26.11

Bases • SSR^-1 ^(kb)	7.048	18.49

% SSR sequence	0.27%	0.06%

Number of repeats	9.96	5.92

Mean number of sequencing reads per SSR, mean kilobases of sequence per observed SSR, percentage of total library sequence contained in SSR motifs, and mean number of repeats per SSR. All data are partitioned by genomic library (i.e. EST library or BAC library sequence). SSR sequences are typically more common in BAC library sequence than in EST library sequence.

### Distribution of SSRs in genomic library sequence

**Figure 1:**
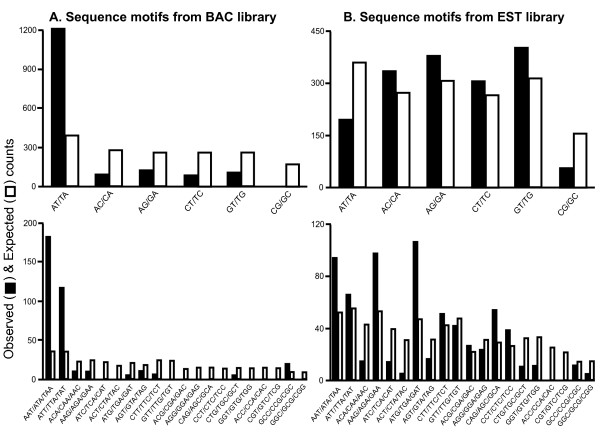
**Nonrandom distribution of SSRs**. Observed (black bars) and expected (white bars) counts of repeats of each sequence motif. Data are grouped by genomic library and by SSR motif size: (A) BAC library dinucleotide repeats and trinucleotide repeats, top and bottom, respectively; (B) EST library dinucleotide repeats and trinucleotide repeats, top and bottom, respectively. Observed values are based on posterior probability distribution of SSR motifs. BAC library SSRs show an excess of AT-rich repeat motifs, while EST library SSRs show a deficit of AT and CG motifs.

To test whether SSRs were more likely to involve a particular sequence motif, we developed a posterior probability distribution of each di- and trinucleotide repeat type (e.g. AG, ACT, etc.) for both the EST and BAC genomic libraries. The analysis could not be extended to include tetra- and pentanucleotide SSR motifs because these comprised less than 2.5% of the dataset and thus were too infrequent to provide adequate statistical power (10 tetranucleotide SSRs in BAC library sequence and 16 tetra- and 2 pentanucleotide SSRs in EST library sequence). The posterior probability distribution was compared to the observed distribution of motifs within SSRs using the chi-square goodness of fit test. In each case, SSRs were found to be significantly non-randomly distributed across motif types (Table 2). This effect was magnified in SSRs identified from BAC library sequence and qualitatively different in EST and BAC libraries. In BAC library sequences, SSRs were strongly biased toward AT-rich repeat motifs for both di- and trinucleotide SSRs (Figure 1). While this was also true of trinucleotide SSRs in the EST library, dinucleotide SSRs from EST library sequence were chiefly characterized by a deficit of repeats containing solely purine or solely pyrimidine bases (Figure 1). Generally, biases in SSR repeat distribution were far greater in BAC library sequence than in EST library sequence. Data were additionally evaluated with respect to a uniform distribution of SSRs across all motif types with qualitatively similar results (i.e. significant deviation from uniform distribution; data not shown).

**Table 2:** Significant departure from expected distribution of SSR motifs

	***X***^**2**^	df	p
BAC (di)	875	5	< 0.0001

BAC (tri)	421	19	< 0.0001

EST (di)	126	5	< 0.0001

EST (tri)	131	19	< 0.0001

Chi-square goodness-of-fit tests showing comparison of observed distribution of SSR motif types to the posterior probability distribution. Tests are partitioned by genomic library and size of repeat motif (i.e. dinucleotide and trinucleotide, di and tri, respectively). *X*^2 ^= chi-square statistic; df = degrees of freedom. All SSRs were non-randomly distributed across sequence motif types.

**Figure 2:**
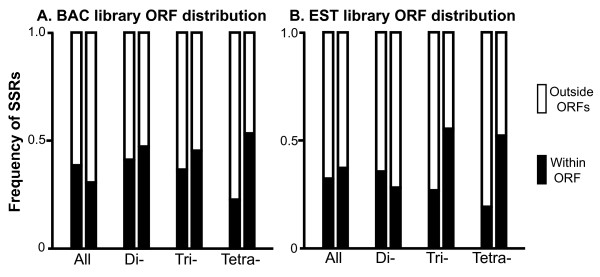
**ORF placement of SSRs**. Paired columns show observed and expected frequencies of SSRs (left and right columns, respectively) within each ORF category and are further subdivided by data partitioning showing pooled data, dinucleotide SSRs only, trinucleotide SSRs only, and tetranucleotide SSRs only presented left to right within each panel. Panel A shows distributions for SSRs from BAC library sequence; panel B shows distributions for SSRs from EST library sequence. Observed and expected frequencies are generally in agreement within BAC library sequence, but diverge in EST library sequence, primarily among tri- and tetranucleotide repeats.

Distribution of SSRs in genomic library sequence was additionally evaluated with respect to placement within open reading frames (ORFs), including all ORFs in any reading frame. SSR ORF placement was characterized as being either within an ORF or outside of an ORF. Comparisons using chi-square goodness of fit tests revealed that SSRs from BAC library sequence were randomly distributed with respect to ORFs, while those from EST library sequence were non-random, particularly among trinucleotide SSRs (Table 3). These deviations from random expectation among EST library SSR ORF distribution are primarily characterized by deficits of tri- and tetranucleotide SSRs and a slight, though non-significant, excess of dinucleotide SSRs within ORFs in EST library sequence (Table 3 and Figure 2).

**Table 3:** Placement of SSRs in genomic sequence ORFs

	BAC library		
	
	***X***^**2**^	df	p
All SSRs	2.17	1	0.140

Dinucleotides	0.64	1	0.423

Trinucleotides	0.71	1	0.401

Tetranucleotides	2.10	1	0.147

			
	
	EST library		

	*X*^2^	df	p

All SSRs	2.09	1	0.148

Dinucleotides	3.54	1	0.060

Trinucleotides	20.2	1	< 0.001

Tetranucleotides	3.46	1	0.063

Chi-square goodness-of-fit tests showing observed distribution of SSR motif types outside of versus within all ORFs to as compared to the posterior probability distribution. *X*^2 ^= chi-square statistic; df = degrees of freedom. Significant deviations were observed only with trinucleotide SSRs from EST library sequence; di- and tetranucleotide SSRs from the same library were nearly significant.

### Distribution of allelic richness in genomic library sequence

Of the 288 putative SSR loci screened for allelic variation in *L. kohalensis *and *L. paranigra*, 35 failed to amplify entirely and amplification was successful for only a single species in two of the markers screened. Of the remaining 251 loci, 9 loci were scored as having at least one individual with a null allele and were dropped from subsequent analyses. In total, 242 putative SSR loci were assayed for allelic length variation (122 from the EST library sequence and 120 from the BAC library sequence, see Additional Files 1 and 2).

**Figure 3:**
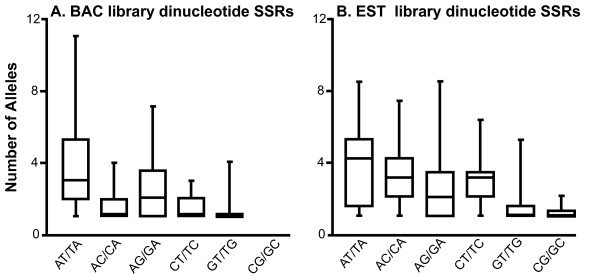
**Allelic richness across dinucleotide SSR sequence motifs**. Medians, interquartile ranges, and maximum and minimum values of allelic richness are shown for each SSR sequence motif type, with GC content of SSR motif increasing left to right across each panel. Both BAC library SSRs (panel A) and EST library SSRs (panel B) generally have greater allelic richness in AT/TA SSR motifs.

Allelic richness was evaluated only for loci lacking null alleles (i.e. those that were successfully amplified and scored in all eight *Laupala *individuals screened) and a simple count of allele number was used to estimate allelic richness. Mean allelic richness was nearly identical for total SSRs from BAC and EST library sequences (μ = 2.98 and 2.78, respectively. Mann-Whitney U test p = 0.837). However, distributions of SSR allelic richness within both BAC and EST library sequence is non-random. Allelic richness was unequally distributed across dinucleotide SSR sequence motifs in both libraries (Table 4). Trinucleotide SSRs were pooled by GC content categories (i.e. 0%, 33%, 67%, or 100%) to increase sample size and no significant effects on allelic richness were detected; however, this likely reflects small within-group sample size (< 8 in most cases) (Table 4). In both BAC and EST library sequence, allelic richness was higher among AT-rich than among GC-rich dinucleotide SSR motifs, visible particularly among AT-dinucleotide SSRs in BAC library sequence. (Figure 3). Although AT-rich motifs were generally overrepresented in BAC library sequence, this pattern was not mirrored in SSRs from EST library sequence. Consequently, this pattern of allelic richness is likely independent of sample size (SSR abundance) in the data set.

**Table 4:** Allelic richness across SSR sequence motifs, ORF positions, and motif sizes

	SSR sequence motif		
	
	***X***^**2**^	df	p
BAC (di)	14.2	4	0.007

EST (di)	13.3	5	0.021

BAC (tri)	1.691	3	0.639

EST (tri)	0.302	2	0.86

			
	
	SSR ORF position		
	
	*X*^2^	df	p

BAC	0.424	1	0.143

EST	2.147	1	0.525

			
	
	SSR motif size		
	
	*X*^2^	df	p

BAC	3.521	2	0.172

EST	1.188	2	0.552

Kruskal-Wallis tests for equality of allelic richness partitioned by origin of genomic sequence. SSR sequence motif compares allelic richness of SSRs with different base composition; SSR ORF position compares allelic richness of SSRs located within versus those outside of ORFs; SSR motif size compares allelic richness among di-, tri-, and tetranucleotide SSRs. Allelic richness is unequally distributed only across SSR sequence motifs and only among dinucleotide repeats.

Allelic richness does not appear to be influenced by placement in ORF regions or size of SSR motif in either BAC or EST library sequence (e.g. di- versus trinucleotide repeat motifs) (Table 4), although the ORF screening methods used here may not have sufficient stringency to fully test this hypothesis.

### Repeat number is correlated with allelic richness

**Figure 4:**
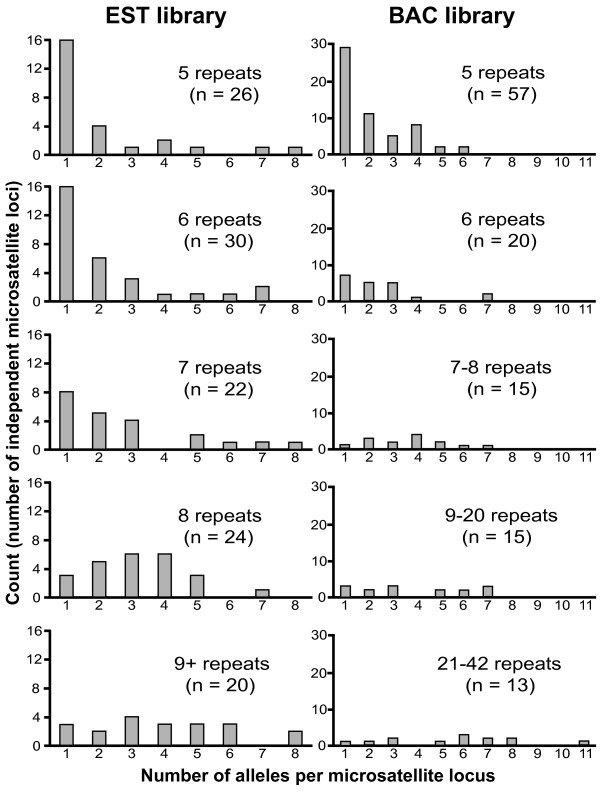
**Allelic richness is concentrated in high repeat number SSR loci**. Distributions of allelic richness within repeat number classes as SSR locus counts. Left column shows EST library SSRs; right column shows BAC library SSRs. Repeat numbers are grouped to increase sample size within each repeat number class.

Allelic richness is significantly positively correlated with the number of repeats present in genomic library sequence SSRs (Spearman correlation coefficient = 0.479 with p < 0.0001 for BAC library SSRs; Spearman correlation coefficient = 0.404 with p < 0.0001 for EST library SSRs). Reference sequence repeat number ranged widely in both genomic sequence libraries. Consequently, data were pooled by reference sequence repeat number within each library to increase sample size within each repeat class (5, 6, 7-8, 9-20, or 21-42 repeats for BAC library sequence; 5, 6, 7, 8, or 9 or more repeats for EST library sequence) and are presented in Figure 4 subdivided by the number of alleles observed at each locus. Allelic richness appears to increase more rapidly with increasing number of repeats present in EST library sequence than in BAC library sequence (Figure 4).

## Discussion

A ubiquitous feature in the genomes of diverse organisms, simple sequence repeat (SSR) loci, or microsatellites, are frequently overrepresented in eukaryotic genomes relative to total base composition [39,40]. Further, SSR loci have been found to vary significantly in terms of repeat length across a broad range of taxonomic scales [13,14,20,38]. In this study, we screened nearly 12 Mb of genomic DNA sequence from the Hawaiian swordtail cricket *Laupala kohalensis*, consisting of both BAC and EST library databases, for all SSR loci. Of the 736 total SSR loci identified, 288 were screened using four distinct families each from two *Laupala *species, *L. kohalensis *and *L. paranigra*, and characterized according to amplification success and allelic diversity at each locus. In our analysis, we found that identities of SSR loci are far more conserved across these two closely related species (i.e. that primers designed for *L. kohalensis *are able to amplify equally well in *L. paranigra*) than might have been expected [41,42]. Despite this observation, these same SSR loci showed significant bias from neutral expectations, both in terms of overall genomic distribution and SSR characteristics corresponding to high levels of allelic diversity.

Consistent with the observation of SSR frequency being positively correlated with genome size and intergenic space [13,16], SSR loci were generally more common in BAC-end sequence than in EST sequence, with a roughly two-fold higher density and double the repeat length. This density is within the range of variation encompassed by *Drosophila *[43,44] and similar to that of the honeybee [45] although this is nearly an order of magnitude higher than that of most bivalves [13]. SSR loci do appear to be slightly less common in EST sequence, but with a haploid genome size similar to that of many bivalves [13] and an order of magnitude larger than most *Drosophila *[38], it seems unlikely that the overall density of SSR loci in *Laupala *is associated with genome size or size of intergenic space in the genome. SSR surveys in the grasshopper *Chorthippus biguttulus *indicate that the large genome size in this species is associated with elevated repeat length rather than density of SSR loci within the genome [46]; however, this does not appear to be the case in *Laupala*, where observed repeat lengths and densities were similar to other insect groups [43-45]. Interestingly, SSR distributions deviated from random with respect to ORF placement only among trinucleotide repeats derived from the EST library, in which variation would not result in a frame shift mutation, and were significantly underrepresented in this case (Table 3, Figure 2). Nevertheless, this deficit of SSR loci within ORF regions in EST library sequence when compared to BAC library sequence suggests that selection on actively expressed regions of the genome, particularly those in frame for translation, likely plays a significant role in the distribution of SSR loci in the *Laupala *genome.

(AC)_*n *_repeats are by far the most numerically dominant of SSR loci in humans and most eukaryotes [47-49]. Contrary to this observation, however, dinucleotide SSR motifs in *Laupala *BAC-end sequences are strongly biased toward an excess of (AT)_*n *_repeats and generally toward repeats with depressed GC base content. This general trend is similar to that reported among SSR loci in the silkworm, *Bombyx mori *[50]; however, the broader generality of the observation is unclear. No parallel compositional bias was observed for SSR loci identified from EST library sequence. This may reflect physical constraints on the structural properties of SSRs (see [15]); however, the contrast with BAC library-derived SSR loci under similar physical constraints suggests a role for selection constraining SSR composition within genic regions. Although SSR loci are well-known to occur within transcribed sequences and "EST-SSRs" are a useful source of molecular markers [30,51], this study suggests that EST-derived SSRs may be strongly constrained by selective pressures.

Allelic diversity has been shown to be positively correlated with the length, or repeat number, of SSRs in many organisms [22,52,53]. Both biased mutation rates and selection acting on allele size have been suggested as mechanistic explanations for this observation [54,55]. Here, we observed SSR loci having between 1 and 11 alleles total in the eight individuals sampled. Consistent with previous studies, allelic diversity was found to be significantly positively correlated with repeat number in library sequence, regardless of library of origin (BAC or EST). Although SSR loci with large numbers of repeats were not necessarily likely to have a high degree of allelic diversity, loci with high allelic diversity did show a tendency to be drawn from high-repeat number SSR loci. This was particularly true of BAC library-derived SSR loci. The two libraries used in this study are drawn from different genomic samples, EST and BAC, and the view of genomic processes shaping SSR repeat-number evolution consequently is different for each library. Biased mutation rates appear to be the primary factor driving allelic diversity in BAC library-derived SSRs while selection on allele size may play an additional role in EST library-derived SSRs. In this latter case, distributions of allelic richness may be constrained by selection against frame-shifting mutations or excessive length of repeated amino acid elements [18] and such markers may be of minimal use, and potentially misleading, for studies requiring the assumption of selective neutrality.

Mean allelic diversity in *Laupala *was nearly equal in SSRs derived from both the BAC and EST libraries, but showed strong effects of SSR base composition. SSRs with low GC content from both libraries were significantly more allele-rich than high GC content repeat motifs. This observation is independent of the numerical abundance of the same repeat motifs, although they may share a mechanistic origin. Different equilibrium microsatellite lengths across species have been attributed to species-specific rates of replication slippage [56]. If the *Laupala *replication machinery is intrinsically more likely to suffer slippage during replication of AT-rich motifs, this may explain both the prevalence of these repeat structures within the genome and their diversity across lineages. Similar patterns of base composition-biased allelic diversity have been reported in *B. mori *[50]. Previous studies have reported strikingly different levels of polymorphism among repeat motifs (di-, tri-, tetranucleotide repeats, etc.) (e.g. [50]); here, no parallel effect of motif size (i.e. di-, tri-, or tetranucleotide repeat) or ORF position was observed, although this may largely be a factor of limited sample size (number of loci) and low stringency of ORF identification used in this study.

## Conclusions

Many applications of SSR loci as molecular markers are contingent on the presence of a number of selectively neutral length variant alleles to distinguish species or lineages [24,25]. Here, we used a combination of *in silico *and laboratory-based analyses to evaluate 288 SSR loci in the Hawaiian swordtail cricket genus *Laupala*, the first such genomic-scale survey in the Orthoptera. Despite a relatively large genome size, SSR loci do not appear to be particularly dense or large in *Laupala*. SSR loci are significantly more common in BAC library than in EST library sequence and are heterogeneously distributed across all potential base compositions, with a deficit of GC-rich repeat motifs. While many SSR loci can be identified in EST library sequence, the likelihood that selective pressures shape the frequency and diversity of these loci may restrict their utility for certain applications. Allelic diversity of SSR loci is positively correlated with the repeat length found in library sequence and also influenced by repeat base composition. In *Laupala*, similar physical structural properties of SSRs and the DNA replication machinery likely contribute to the elevated abundance and allelic diversity of AT-rich repeat motifs, suggesting that future screens of Orthopteran molecular markers may benefit by focusing on such SSR motifs. Although allelic diversity profiles are similar in both BAC and EST library-derived SSR loci, the generally higher frequency of SSRs, larger number of repeats within those SSRs, and reduced likelihood of strong selective constraints relative to EST library-derived SSR loci make BAC library sequences far better *in silico *sources of SSR loci in *Laupala *and likely other developing model systems as well.

## Methods

### Sequence data and analysis

14363 EST sequences, comprising 10.17 Mb, from *L. kohalensis *were downloaded from the DFCI Cricket Gene Index http://compbio.dfci.harvard.edu/tgi/cgi-bin/tgi/gimain.pl?gudb=Cricket. An additional 1.71 Mb of unpublished BAC-end sequence, also from *L. kohalensis *were evaluated in parallel (library construction and sequencing by Amplicon Express, Pullman, WA). Files were compiled using the program BioEdit (Ibis Biosciences, Carlsbad, CA; available at http://www.mbio.ncsu.edu/BioEdit/BioEdit.html and redundancies were eliminated based on sequence identity. In total, 11.88 Mb of genomic library sequence from *L. kohalensis*, averaging 703 bp per sequencing read (674 and 708 bp per sequencing read for BAC and EST libraries, respectively), was available for analysis.

Both genomic libraries were screened for SSR motifs using the Msatfinder script implemented in PERL ([57]; available at http://www.genomics.ceh.ac.uk/msatfinder and all SSRs having repeat motifs of two or greater base pairs and greater than five repeating units were identified. Only simple, perfect repeat motifs were considered; compound and imperfect repeat structures were not included in this analysis. Primers flanking each SSR were designed using Primer3 ([58]; available at http://frodo.wi.mit.edu. Primers could not be designed for terminal repeats (i.e. those SSRs falling at the end of a sequencing read) and all such SSRs were subsequently dropped from the analysis. All primers were designed to produce products ranging in size from 200-350 bp with an optimal T_m _of 60°C. Maximum self-complimentarity was set to 8.00 (3.00 at the 3' end) and maximum self-priming was set to 12.00. Program default settings were used for all other parameters.

The location of each identified SSR with respect to open reading frames (ORFs) was determined using the GenBank Open Reading Frame Finder http://www.ncbi.nlm.nih.gov/gorf/orfig.cgi and categorized as being either outside of all ORFs or within one or more ORFs. SSRs bridging an in-frame to out-of-frame boundary were discarded from subsequent analysis.

### Animals

Four individuals each from two species of the Hawaiian cricket genus *Laupala *were used to screen SSR loci for length variants. The two species, *L. kohalensis *and *L. paranigra*, are both from the Kohalensis species group and show less than 0.1% nucleotide sequence divergence at nuclear loci [31,33]. A single *L. kohalensis *male was drawn from each of four lines, collected from two different sampling localities each in the fourth laboratory generation (Pololu 2006 female #1, Pololu 2006 female #11, Kupehau 2006 female #6, Kupehau 2006 female #1). A single *L. paranigra *male was drawn from each of four lines, all collected from the same sampling locality (Kaiwiki 2006 #7, Kaiwiki 2006 #8, Kaiwiki 2006 female #1, Kaiwiki 2001 LP1), with the first three in the fourth laboratory generation and the final line in the eleventh laboratory generation.

All individuals were sacrificed by decapitation using a sterile razor. Thoracic and abdominal sections were archived in 100% ethanol, while the head was used for DNA extraction using the DNeasy Blood and Tissue Kit (Qiagen Inc., Valencia, CA) according to manufacturer's specifications. DNA concentration in each sample was evaluated using a NanoDrop spectrophotometer (Thermo Scientific, Wilmington, DE) and each was diluted to a working stock of 10 ng•nl^-1^.

### Screening of SSRs

288 identified SSR loci were screened for repeat variation in the eight animals listed above. PCR reactions containing 1X PCR DyNAzyme II buffer, 0.05 U•μl^-1 ^DyNAzyme II (both from Finnzymes Inc., Woburn, MA), 0.2 mM dNTPs (New England Biolabs, Ipswich, MA), 1 μM each forward and reverse primer (Sigma-Genosys, St Louis, MO), and 0.5 ng•μl^-1 ^genomic DNA template were performed in 20 μl volumes. A denaturing step of 2 minutes at 95°C was followed by 35 cycles of 95°C for 30 seconds, 56°C for 30 seconds, and 72°C for one minute and a final 5 minute elongation step at 72°C. Although these conditions did not work for all 288 primer pairs, no attempt was made to optimize conditions for the minority that failed. Primers are presented in Supplementary Table 1.

All PCR products were evaluated for length variants on 4% 19:1 polyacrylamide:bis-acrylamide gels (SequaGel Sequencing System, National Diagnostics, Atlanta, GA). Each gel was pre-run for 15 minutes at 60 W prior to loading samples and 100 bp ladder (New England Biolabs, Ipswich, MA), then run for 3 hours at 55 W. Bands were visualized by silver staining and scored by hand. As precise determination of band size could not be attained, scores reflected number of unique alleles per SSR locus.

### Statistical analysis

All statistics were performed using the SPSS11 statistics package (SPSS Inc., Chicago, IL). Mann-Whitney U tests were used to test for difference in repeat numbers present across libraries. Chi-square goodness-of-fit tests were used to compare observed SSR distribution within libraries with respect to (A) even distribution of SSRs across all sequence motifs (B) the posterior probability distribution of repeat motifs, (C) an even distribution of SSRs internal and external to identified ORFs, and (D) the posterior probability distribution of ORF inclusion. Posterior probability distributions were calculated separately for each genomic library, BAC and EST, respectively. The posterior probability distribution for SSR motif composition was calculated based on the total frequency of SSR motif within a complete genomic library. Correlation between SSR reference sequence repeat number and allelic richness was performed using a Spearman rank correlation. Distribution of allelic richness with regard to (A) library sequence repeat number, (B) placement within versus outside of ORF sequence, and (C) size of SSR motif (i.e. dinucleotide repeats versus trinucleotide repeats versus tetranucleotide repeats) was evaluated using Kruskal-Wallace tests. Similarly, the posterior probability distribution for SSR placement within ORF regions was based on the distribution of ORF sequence within those library sequences containing identified SSRs. To eliminate the possibility of a sampling bias influencing estimates of allelic richness, any loci with one or more individual failing to amplify were removed from analyses using allelic richness estimates. Consequently, all estimates of allelic richness were based on an equal number of individuals (4 *L. kohalensis *and 4 *L. paranigra*).

## Abbreviations

SSR: Simple Sequence Repeat; ORF: Open Reading Frame; DNA: Deoxyribonucleic Acid; EST: Expressed Sequence Tag; BAC: Bacterial Artificial Chromosome; PCR: Polymerase Chain Reaction; bp: base pairs; Mb: megabases (=10^6 ^base pairs)

## Authors' contributions

CKE designed the experiment, collected all data, performed data analysis and contributed to writing of the manuscript; KLS contributed to interpretation of results and writing of the manuscript. The final version of this manuscript was approved by both authors.

## Supplementary Material

Additional file 1 **Laupala SSR primer and repeat data from BAC library sequences**. Table showing all SSR loci screened in this study for allele number. Includes: SSR sequence identifier, primers, reference sequence repeat number, and observed allele number for SSRs derived from BAC library sequence. Click here for file

Additional file 2 **Laupala SSR primer and repeat data from EST library sequences**. Table showing all SSR loci screened in this study for allele number. Includes: SSR sequence identifier, primers, reference sequence repeat number, and observed allele number for SSRs derived from EST library sequence. Click here for file
