# Supplementary material for: Mining non-model genomic libraries for microsatellites: BAC versus EST libraries and the generation of allelic richness
Source: BMC Genomics. 2010 Jul 12;11:428. doi: 10.1186/1471-2164-11-428 (PMC2996956; doi:10.1186/1471-2164-11-428)
Supplement: Additional file 1 — Laupala SSR primer and repeat data from BAC library sequences. Table showing all SSR loci screened in this study for allele number. Includes: SSR sequence identifier, primers, reference sequence repeat number, and observed allele number for SSRs derived from BAC library sequence. [file 1471-2164-11-428-S1.PDF]

Locus	F primer	R primer	Repeats #	type	Allele #
EH629318	CATGATGGACGCAGTTATGG	ACTCTATCCTCACCCCGACA	7	AG	1
EH629457	AACTGAAGGAGAAAAAGAAACCTG	AAAAAGGGAAAAGGCAGGAG	8	AC	4
EH629650	TCATTTTGCAATGTCTGTTCTT	GGCACTGTTTACTTGGATGTCA	7	ATC	7
EH629699	AGCAAATTTTCCTGGCTCAA	ATCTCTCCTCGGTGCTTCAA	8	AGA	3
EH629705	TGTGGGTATTGGGGATCCTA	CACTTCTCGGCCAATACACA	8	CT	-
EH629741	CATCATGCAAAAGGTTCACTG	TGCAAACTGCGAAAGACTTG	8	TC	3
EH629893	AGTGAATGCGGGTGTACCA	CACTTCGGGTTGATCGAAAT	8	AT	4
EH630110	ACGTTTTGTCCCTTCCTTCC	TGCACCTTTTGAAACTGCTG	12	TTC	8
EH630221	CCTGTGGGGACATTGAGAGT	TAAATCCCAACACAGCACCA	8	AG	2
EH630470	CATGATGGAGCGTTTCTTGA	CCTCTTGCATATCGGGTGTT	7	AAG	-
EH640112	TCTCAAGCATTGGTGCATTT	GCTCAGGCCAACTTCGTTAC	6	TTG	2
EH630601	CTTCAGAACGGAACAAACTGC	CCGTCTTCGGCATTAGTTGT	8	AGG	7
EH630637	AGACCGAAAGGAAACGAGGT	AAGCGCTTGCCTAAACGATA	10	CT	-
EH630642	ATCGTCGCTCCAACGTAATC	GTTCACGTCGTAACCCCAGT	10	GA	1
EH630651	ATCGTCATTTGCAGTCACCA	CAGCACCACATCAAAAAGGA	8	AG	4
EH630735	AATGTTGGCTCTGGGATCTG	CACGGTTTGTTTACGCAGAA	8	AC	2
EH630768	TCGTTATTGCCCACTTGTTG	TCTCAAGAGAATCCACTCACCA	8	AT	-
EH630969	CCACCCCCTCATTTTTAGGT	AATTTGGACAGGGCATCATC	12	GA	8
EH631041	CAGAATTGGACTGGGGAGAG	CCACTGTGAAGGTATCAGAAGC	7	TC	5
EH631204	AACGTCGACAGCACTGAAAA	GTGTAGCGAGAAACGCTGTG	8	CT	1
EH631211	GCGCTCGGTCATCATACTTT	GGCAGCCTTCTCATGGTAAA	11	AG	3
EH631312	GGCGTGGAGTATGAATGTTG	GGAAGAAATCAATATCCCACCA	6	TTTG	-
EH631475	ACCCACACGAACGTCCTTAG	TGGTTGTAATCGTGCAGCATA	17	AG	-
EH632048	GGGATGGATACCATTTGCTG	TTCCTCGCCCAACATATTTC	7	CTCA	5
EH632101	CCACTTATGAACGTATTCGCAGT	CAACGTTTTGTAACGGTTGGT	9	GA	2
EH632301	TTGGTATTTGCCAGATTTTTCA	GGTCCCTGTTAGCTCAGTGC	11	TC	4
EH632421	GCAGGAGGAAGGAACAAATG	TGATCGAAACGTTGGCTATG	8	AGAA	-
EH632427	TCAGATGTGCCACCCTGTAA	TCCCCAGCCACAATTCTAAG	9	TC	-
EH632493	AACGAAATCCACGAAACTGC	TTGTTCTTGGAGGAGGTTGG	13	GA	-
EH632516	AACGAAATCCACGAAACTGC	TTGTTCTTGGAGGAGGTTGG	13	GA	4
EH632720	TACCACGCGAAACACAAAAC	TTGATTTTGAGGCCTTTTGG	9	AC	2
EH632777	CAACAACACCAGCATCACC	CGACATACGGTCTGTGCAGT	9	CAG	-
EH632830	TGGGGTACTCCGCAAGTAAG	AGATGACGAGAGACGCAACA	7	AG	-
EH632923	TGTCGACATGAGCCGTAAAG	TCCTTCAAAACTGACAGCAATG	7	AC	2

EH633131	TTTTACTTCGGCTCGGAATG	AGAGCGACGTAAACGCTGAG	7	GAA	1
EH633325	TTTTCAAAGAAGGCAAGAAACC	TCGTGGAAAAGTATTGGAACC	7	AT	2
EH633595	AGCAGAAACTCGTGCATCCT	AAGTGCTCGTTCCATTCCAC	8	TC	2
EH633743	AACCGTTCATTTCGAAGGAC	GTGCACAACCGGCTATAGGT	12	CA	4
EH634066	TTTCTCCAACTTTGGCGTGT	CTTGGCAGTCAAGGAGAAGG	7	CT	3
EH634219	AGCCTCGCAGAGGTTGTTAG	ATCTTACGCAGCTCCTCCTG	7	CA	-
EH634430	CGGCAACTAACCCCTACTTG	AGGTTACGCGTGCTGTTTCT	8	AC	5
EH634451	AGTGAATGCGGGTGTACCA	CACTTCGGGTTGATCGAAAT	8	AT	5
EH634620	CCTGTGGGGACATTGAGAGT	TAAATCCCAACACAGCACCA	8	GA	-
EH635272	AACACCGTTGTGTGGTTTCA	CGTCGCGGTATGTTTTTACC	8	AG	3
EH635281	TTTGTGGCTAATGACGGTGA	CTGAAAGGCACAAAGGGAAA	16	CTT	6
EH635714	TCGAACTCTGGAGCTTCCTC	CTTTAGCCCCACGATGAGAA	8	AC	4
EH635961	CGAAACAGCAGACGACTTCA	GCGATGAACGGAACCTGTAT	8	CA	2
EH635970	AAAAATCGAACACAGGGTCA	AATGTGAAAATCACGCACCA	7	GA	2
EH636295	TCCGCGAAATAACTAAGTCCA	TCTACTTGAACATCTGCTTGTGC	8	TC	2
EH636512	TTGGATGCGTCTTTGTTCAG	ACGTATCACAAGCGCACAAG	7	GT	2
EH636571	GATGGTGGATTCCATGTTCC	CCAGCGTCAGAGGTAGAAGG	7	CTC	-
EH636584	ACCGCAAGGTCCTAACAGAG	CGTCAAAAGGCATAAAAGAAATG	7	GAA	-
EH636688	AGTGAATGCGGGTGTACCA	CACTTCGGGTTGATCGAAAT	8	AT	-
EH636816	GCAATGTAAATCGACCTCAGC	TCGTGGAGCAATGTTTTTGA	7	GT	1
EH636820	AGTGAATGCGGGTGTACCA	CACTTCGGGTTGATCGAAAT	8	AT	5
EH636896	GGTCGTAGACCTCGTTCCAA	GATCTCGACTGGGACCTTGA	8	CAA	4
EH637037	CGAGAAAGGGAACATTCCAA	GGGAGGTGAACTTGTCCGTA	7	CT	-
EH637244	GCAACAGCAGATCCAGAACA	GACTGTACGGAGGGATCCAA	7	CAG	-
EH637321	GAATTAAAGTGCCGGCAAAA	CTGAAGGAAAACCGGAGAAA	7	TC	-
EH637392	CTCGTATCTGGGAGGCAATC	GGGCAGAGACGAATTTTCAA	5	CT	3
EH637439	GAAGGAAACGGGAAGGAAAG	GAGAGACATGACGGACTTGGA	8	TC	-
EH637709	AGAACTCGAAAGTCGCAGGA	ACCCCTCCCTATACCCACAC	9	AG	5
EH637849	CGCCGCAAATAGGTATTGTT	TGAAATCGGGGTAGGTTGTT	8	GA	1
EH637908	GCAAATGTGTGGCATGAATC	CCAGCTCGAAGACCCATTTA	5	ATTT	1
EH638016	CAAAACCTCGGGTACTCAGC	CCACGCTAATTGTTTGTTGG	9	CT	3
EH638105	ACAGAACTTCGCCATTGGTC	AAAATCAAACTGAAAGAAATGTCA	8	AC	4
EH638173	AAGCTCGTGCTGTGGTTTCT	TCTTCCGACGATCATCACTCT	7	GA	1
EH638225	CAGTCATCGTACTCGGCAAA	TATGGCTTGCGCTTTCTTCT	10	GA	6
EH638391	TCCGGTTCGATTTTTCTGTC	CGTCGTTGTTCACAGATGCT	9	TG	5
EH638469	TGGCACTTACAATTCCGAGA	TCAAGAGCCAAGTACTTCAGGA	13	CA	3

EH638872	AGAGGATGCGATTTCCCTCT	TCTGTCACGGACATCTCTCAA	7	GA	-
EH639160	GCGCTCTAATGACCTTGACA	TTGAACCAATCCGCTCAAAT	7	CA	2
EH636823	ATACGACGTCCTCGTGTTCC	GAGAGGACGAGGTCACGACT	6	TTG	1
EH639255	TCAACACGAGTACTAACGCACA	ATCCAGTGTGGGGCATGTAT	8	AAT	3
EH639639	AGTGAATGCGGGTGTACCA	CACTTCGGGTTGATCGAAAT	7	AT	-
EH640099	TGCCATTTCATATTCGTTTCG	TGCACGAACGTTGGATATGT	10	TG	1
EH640113	GGAGAGCCAACATGACACCT	GCAGTTTGCAACAGCTCACT	9	AG	3
EH640483	GCACGTAAAGTGGGTTTTGC	GGCTTATTCGTCAACGAGGT	13	GT	-
EH640545a	TGAACTTGCCCTCGAAAACT	CACCATATTCCTTCCCGATG	7	TTA	1
EH640545b	TTTTCCACGGATGTGTGTGT	AGTTTTCGAGGGCAAGTTCA	7	TTG	1
EH640701	AGTTGACGAAGATGGGGATG	CCGATGAACATGCTGCTTTT	7	GA	1
EH640771	GCCACTGGTTCATACGCTTT	GCTGCCTCTAAACCTGCATC	11	CT	5
EH640975	GGGGGTAGCCATCCTAATGT	TACTCTTTCCCTCGGCTTGA	9	CT	6
EH640999	TGGCGTGAATGAAGGTACAA	TTAAAATGTCGCCAACAGCA	8	GT	1
EH641073	GCAATGTAAATCGACCTCAGC	TCGTGGAGCAATGTTTTTGA	7	GT	-
EH641286	CAAACGAAAGGGGATTCAGA	CGTTTGCGATGCTTCTTCTT	5	AAG	-
EH641442	CGGAATTGATGGGATACGTC	TACCCGCCTTTACCTCCTCT	7	AG	1
EH641735	CTTCTCTGAACCCCGTTTGA	AATTCTCGATTGGGGGAGAG	7	GA	3
EH641963	CCATGTGGCACACTGCTTAC	CGAATGACGAATGTCTGGAG	8	CT	3
EH642092	TGGACAAATAGTGCGACCTG	TTGGGAGCCGTACTGTCTAAG	7	AG	3
EH642266	AGCACTCACTTTTCCCCACT	GCTTACGTTGCCAAAATGGT	10	TG	1
EH642437	CAGGAGACGACCAAGTGTCA	GCGATGGTGCCTCTTTTAGA	7	GCA	8
EH642443	CAGGAGACGACCAAGTGTCA	GTGGCGATGTGCCTCTTATT	7	GCA	-
EH642875	ATAAGCCCGCCTCAACTACC	CCCGGAAAATAGTGACAAGC	7	ATA	6
EH642982	CTCGGGCACGATGTTTATTT	GTTTCCAGAGGTCGCCTTTC	7	CA	3
EH643045	ATTGAATGGGCTGCTAGTGC	GCACAAATGGAAGGCATTTA	8	GT	3
EH628899	TGCCGAGAGACAGTTGAGAA	GAGGAGGTGGATGATGCCTA	5	GC	1
EH628906	TAAACGCCCCAGCTCATAAC	ACCTCGCGTTGATTATGTCC	5	TA	7
EH628935	GAGGAGGTGGATGATGCCTA	TGCCGAGAGACAGTTGAGAA	5	CG	1
EH628943	TACCGACATCAATCGGTGAA	CAGGCATTTCTACCCCACAT	5	TG	1
EH628971	ACCTCGCGTTGATTATGTCC	TAAACGCCCCAGCTCATAAC	5	TA	8
EH629004	AGGTCGGGGAAGAGTTTTCT	CAACTTGCCGACTTCCCTTA	5	AG	1
EH629034	GCGGGAGGATCATTAACAAA	TAACTTGCGTTCGCTTTTCA	5	TA	4
EH629080	TGCCAAAACCAAAAGAAACC	TGGGACACAGTCAGTGGGTA	5	TG	-
EH629101	TGTGGGCTAACGAACCTCTC	CTGGGAGCCAGTCTTCACTC	5	CTG	-
EH629119	TCCCAATTTTTGCTGAGAGC	TGGAACGATTCCAAAAAGGT	5	AT	1

EH629129	GGATTGGGCAGACCTTACAC	GCATCAATCCATTGCAGAAG	5	GA	1
EH629248	AAAAGTTTGGACGAACTTCAGG	TGGGAGTGGCTCAGTTTTCT	6	TA	1
EH629250	TCAATTTGCCATTGACATCG	TTTTGTTTTTCATGAGGACTGC	5	AG	1
EH629252	TGGTCAAATTCATGCCATTC	GCTGGGAGAAAGGTATTGACC	5	AC	1
EH629278	GTTGATGCGCCTTCAAAAAT	TGGAAATGGGAGAGTTCCAC	5	TG	1
EH629326	CGTGGAAGTGTGAAGTTTCG	GGTCCCAAAAACTGTCATCG	5	TG	1
EH629336	CAAGACCGTCGGATTCTGTT	GGACCAACTCCCATAATTGC	6	CG	2
EH629416	AAAGATCAGCAAGCAAAACAA	CCCATTGGCTTAGACTGGTG	6	CA	3
EH629422	AACACCAGCAAAATTTCAACAA	TGCATTGTTTTTCGGTGGTA	5	CT	1
EH629437	CATCATGGAAAACGACTCCA	ACTCGTGTGCGATGAACGTA	6	TG	1
EH629452	GCGTCGAAAATGGTATGGAC	TGGTTTTCTGCTTTGATGGTT	5	CA	4
EH629502	TGCATATATGGTAACGAATGGTG	GAAGAAACGGGCACTGAAAA	6	GT	1
EH629560	TGTTTCCCCTCTCCTCTCCT	AAGCAGTTCCACGGAAGAAA	5	GTCC	1
EH629583	AAATTTTGAATCCGCCTCTG	AAAAGGTTTGAGAATTTTCATCG	5	AAG	1
EH629606	GGCGATTGCTTCAAGATGAT	GACGTTCGACCCCTATCAGA	5	CT	2
EH629614	TTACCCCGCTATTCCTCCTT	CTTCAAGCCGCTCTCACTCT	5	GAC	2
EH629632	CCATTAGGCCAGGAAACAAA	ATCCGCCGCAGAATTTAATA	5	TGG	2
EH629677	AGGATTTGGCACGTCATGTT	TCAACAATGCATAGGGGAAA	6	AT	1
EH629678	GAGTCCATTACGGCTGGGTA	CTCTTACAATGCGTCCGACA	5	GA	1
EH629785	AGACATCCTGCCAACCAATC	CAACATGGAAACGCAAAATG	5	TGA	1
EH629789	GCGCTTACATTCACTCGGTA	CCTCAGCCCAAAATTCACAT	6	AAT	7
EH629889	AACTCCCCTGAAGCCAAAAT	CAAGAAAAGTTGCACGCTCA	5	GA	-
EH629892	CCAGTATCGGGACACCAATC	GTAAAGAAACCGCCCATACG	6	CA	3
EH630043	TTCCCTGTCGCTTCAATTCT	CACGAAGAGGTGAGACAGCA	6	TTC	4
EH630126	AATCCGATGTCGCTTACCAT	ACGCTAACACCTGAGCCACT	5	AC	2
EH630166	AGCACCCATGCATAAACACA	AATCAATGCCAGTCAAGCA	6	TG	1
EH630207	AGAAGTCACGGCAGAAGCAT	ATAGCTGTGGTCCGGTGTTC	5	TG	5
EH630210	TCCTGATGAGGTCCCAGAAG	TCCATGCCAAGAAAGATTCA	5	AG	-
EH630600	TGCTAAGCAAGTTGGGGAAG	CTCCACAAGGCTTCCACTGT	6	TGA	1
EH630723	CGGTGACTTCTGCCTCTCTT	TTGAGGGACATCGCAGAAAT	6	CA	7
EH630828	TGCATCTCTCACCTTTTTGC	TTCGATGGGAATACGACACA	6	ATTT	1
EH630862	TCGGTGGTTTTTGAGGAAAG	TTGCAATCATCGTCTTGCAT	6	TAT	2
EH631019	TGAGAGGAACCCGATTGAAG	ACCACAACATTGGCGTGTAA	6	GAT	1
EH631094	GAATACCCACAAGCCCAAAA	CTTCCACCCACCTCCTATCA	6	AC	1
EH631253	AGTGGTCATCCTGTGTGTCG	GGGAGAGGTTTTCTCCGGTA	6	GC	1
EH631355	GGCTATACCGTGCACCAACT	GATTGCATAAGGTGCGGTTT	6	TC	2

EH631460	CTGCTCCACATGCACATTCT	AGGGATGGAGAAGCCGTACT	6	TC	1
EH631789	GAAGAAGTTTGGAGGCCACA	CCACAAGGCTTCCACTGTTT	6	GAT	1
EH632064	GAAGAAGTTTGGAGGCCACA	CACGTTTTTCATCCCAGTCA	6	GAT	1
EH632416	CTTCCATTGATCCACCACTGT	GCAAACTCGGGAGGATTTCT	6	TG	1
EH632545	TACACTGGTCGACACGAAGG	CGATCGACTTGCTCATCGTA	6	AAAT	2
EH633025	CCAGCACTGAAAATACCATCC	CCTGCATGACAGCAACAAGT	6	AT	2
EH633154	TCAACATTCCTCGGAATACCA	AAAAATGGTCAAATATGGCTGTG	6	AG	5
EH633198	ATCACAGCTCGGCGTCTATT	TCGCGAGAGTAACCACACAC	6	ATC	3
EH633848	ACTGCACTATCCGGCAATCT	TTCGTTGTTTGCGTTCTCTG	6	CA	1
EH633868	ACTGCAGTGCATGGTAGTCG	ATTCCGCTCACGTCTGTACC	6	GAA	6
