# Supplementary material for: Mining non-model genomic libraries for microsatellites: BAC versus EST libraries and the generation of allelic richness
Source: BMC Genomics. 2010 Jul 12;11:428. doi: 10.1186/1471-2164-11-428 (PMC2996956; doi:10.1186/1471-2164-11-428)
Supplement: Additional file 2 — Laupala SSR primer and repeat data from EST library sequences. Table showing all SSR loci screened in this study for allele number. Includes: SSR sequence identifier, primers, reference sequence repeat number, and observed allele number for SSRs derived from EST library sequence. [file 1471-2164-11-428-S2.PDF]

Locus	F primer	R primer	Repeats #	Repeats type	Allele #
LKH-001_A01_F	GGCAGTGCCAAGATCATCTAC	CATAAGTCGCGCACTCAAAC	5	AT	4
LKH-001_A03_R	TGTTGGGGAGAATAATGAACG	TTGGCAACAACTCAAAAACG	6	TG	1
LKH-001_A07_R	AGTCGAAACTTTGGCAGTCC	CCGTGTTGATCGTCAGAGAG	5	AATA	4
LKH-001_A12_F	AGGCATGCAAGCTTTAGGAA	GAAGAGACGCACCCTCTGTC	8	ATA	4
LKH-001_B12_R	TGATGCGAGTTTGAATGAGG	CGAGCACCCGAGTATTCAAG	7	ATT	7
LKH-002_B16_F	CATGGCATCTGCAAGCTAAA	AGATCCCCTTCACCTCACG	5	AAAC	-
LKH-002_B18_R	CAATTTCAATAGGCCGCTTT	AAAGATGCACTAAAGGATGCTACT	10	ATA	6
LKH-002_C12_R	GAGGACACGTTACGCCAAAT	TGATTCCGGTTTCTTTCAGG	30	TA	7
LKH-002_C15_F	ATCCGTCCTTCAGATTCGTG	CAAGCAAATCGATGACGAGA	5	ATT	6
LKH-002_C21_R	AGTTTCGGCGGTAGAATCAA	GCGGAACTGATGAAGGAGAT	35	TA	8
LKH-002_D03_F	TCCGTTACCTCACACCTTGG	CATGTGCTTGTACCCATATCC	5	ATCT	5
LKH-002_D11_R	GAAGGCGTCCACCTGTAAAA	TGCCCGGCTGAAAGATATAG	29	AT	3
LKH-002_E02_F	GCAGGCATGCAAGCTTTATC	CTGCATGAGGAAAAACACGA	5	TA	1
LKH-002_E02_R	TCCCCTACCACACATCCTGT	CGGAGAGAGGTATGCCGTAA	5	ATA	3
LKH-002_E20_R	CGCATGAATGTTCGAGCTAA	GTCGGCGTAGTTGAAGGAAA	34	AT	-
LKH-002_F09_R	GGTCCAGCGGAGACTAAATG	GGCAAACGTGAGGTAGAAGC	7	ATT	6
LKH-002_F23_F	GCGAAGTCATACTAGGCTCCA	CGGTTGTTGGGAAACTGACT	6	CA	1
LKH-002_G12_R	GGAATCTTTGGGGCAGTTTC	TTTTGATTGGTTGCTCACGA	5	ACAA	-
LKH-002_G20_R	GAAATGCTTCCGGAAATGAA	TTTGAAACACACGAATAATTTTACA	5	GAA	2
LKH-002_G24_R	TTGACACGGGACAGAAGTTG	CGGAGTGGCGTTTGTATTTT	5	TAAA	4
LKH-002_H13_F	ATCCCATCAATTCCGATCAA	ACATGCCATCATCCAAAACA	5	TAA	4
LKH-002_H15_R	TGCAGTGTCCTGGAGAGTTG	GCAGTCTGGACGAACATTCA	7	CT	2
LKH-002_H19_R	CTCTTGGAATGTTTTCCTCCA	CGAAGTGTTGTGAGAAATTGTGA	5	AT	1
LKH-002_H22_R	CGCGAACCGGACTATAAAAA	TTGAAGCGGTACCACACATT	5	ACA	1
LKH-002_I12_F	AGCCAAGTCGAAGCCAACTA	TTGTCCTCCACGTGGTCATA	28	TA	1
LKH-002_I14_F	AAGCTTCATGCTGCTCCATT	GACTGCTTCTAGGGTGCTAAGG	8	ATT	-
LKH-002_I21_R	ACTCCATCACTCGCTCTTCC	CGACATCTCTTCATTCGCTTC	5	AT	2
LKH-002_J12_F	CTCGAGCAAACCGTTGTGTA	TATTTTGTGGACGGCGTGTA	5	GT	1
LKH-002_J17_R	GGTTGGAGGTCAACAATAGCA	ATCCTTCTTTGTGCCCTGTG	5	GA	1
LKH-002_K13_R	GTTTCATCCAACGACGGAAT	TGATAATTCACGTTGCATGTGT	6	GA	3
LKH-002_K24_F	GCAAGCTTGTATTAACAGAAGGAA	GCATCGTGTCGTTCATTTCA	6	TAA	1
LKH-002_L01_F	CATTTACGGCGACTGGGTAG	TTCCATCGTTGACAGATCCA	5	CA	2
LKH-002_L10_R	ATGGGACTCCAACGAGTGAG	ACCATGTGGGAGCCAAAATA	6	TTA	1
LKH-002_M08_F	CGTGTCGCGATTAACTGTGT	AGGTCGCACGGATAAATGAC	6	AT	2

LKH-002_M11_R	ACAGTACGTTGCCGGTTAGG	CATCACTATTTCGCCCCAAT	7	TTA	5
LKH-002_O18_F	GATTGTGTTCAGGGGCAGAT	ATTTTCGCTTCGAAAGTCCA	9	GA	1
LKH-002_P11_F	AACAACGCCGGGTAACCT	CAGTTCCCTTCTTCAGTAGCA	5	GA	2
LKH-002_P18_R	GATTGGAATTGATGGGATGG	CGTCAGAGAAGGGTGGAGTC	15	AAT	7
LKH-002_P20_R	TCCTGTTATCCCAACCTTGC	TGACTTCCGATGTGCTTGAC	13	ATA	6
LKH-003_A10_F	GTGCGAATCGTTTTGTTGTG	TCCACGTGGTCATATGCAGT	5	TC	1
LKH-003_B17_R	CTCCAAGATCTGATGAAGACGA	CGTCAGGTATTTGCCACTTACA	5	ATT	2
LKH-003_C10_F	TGACGAAGCTTGTCGTTCAT	AGGGGACTTCCTCGTCAACT	5	TG	-
LKH-003_C21_F	ATTGAACCCGCCTTCTGAG	TTAACGCTTGGGATGAACAA	39	TA	6
LKH-003_D12_F	TCGAAAAACACCCTTCATGC	ATGTTGGCAATCCCTAGTGC	5	AC	4
LKH-003_D12_R	TGAAACATGGTAGGCGCTTT	TCAAAATTTACACATTTTTGTTCCA	5	GGAA	2
LKH-003_D13_F	GCTGCTTCTCTTTGGAATCG	TGCGAACAGAGTTTTGGTCA	20	TA	3
LKH-003_F05_F	TGAAGGGACAGCTGTGAGTG	GGCAAAGAAGGAGGGAAGAC	8	CT	3
LKH-003_F13_R	CCCTGAGTGTTCGTTCCATT	TGCCACAGTCCACAAATCAT	6	TAA	1
LKH-003_F14_F	GATCATTAGGCTGCAATCTGG	TGGATTACGATGTTTTCACAAT	40	AT	7
LKH-003_F18_R	TTCGATCTTCGTCTGTCATGTT	TGCTCCTCGTGTGAATTGTC	6	TC	-
LKH-003_H14_F	CCCTTTCCATTTTTCCCACT	TAGAATCCGCCTCTGCCTTA	6	TAT	1
LKH-003_H23_F	GCATGCAAGCTTCAATAGCA	TGTGGTATCGTTCTCGTTTCA	10	AG	3
LKH-003_J09_F	GAAGGAATGCATCCCAATGT	CCGAGCGATTTATCAGCACT	17	TA	5
LKH-003_K08_F	CCAAATCATTCAAGCGGTTT	GGATTCGCGAGTTCGACATA	7	TA	2
LKH-003_L06_R	TGTTGTCGCTTCTCACCAAG	AAATCCGAGCCAGGATGG	7	TC	1
LKH-003_L14_R	TAATGTCAGCAGCCACCAAA	GGTTTCAAGGGATGATCCAA	5	AG	1
LKH-003_L17_F	CAACCGTCCTTCGTTCAAAT	GCTCCAGTGATGCTCGGTAT	5	GAA	5
LKH-003_M10_F	AGGAGGAATTTGACCCCAGT	GGGCCCGTTTCACATAAGTA	36	TA	11
LKH-003_N12_R	AATTGCTCCTCACCATCTGA	TTGCTCCTCTTTACTTTGTGAGC	5	TA	1
LKH-003_O04_F	GTTGGGGTTCACGGAGATAA	TGGTGAACAGGGACATGCTA	41	TA	8
LKH-003_O05_F	AAAAAGGCAGCACCATCTTG	GTGCTCTACGCGTTCCATTC	5	CA	-
LKH-003_O13_F	AGGCAAAGCCATACATCCTG	GATGCCTCCAAAAGTACACACA	6	TTA	3
LKH-003_P21_F	TCCTCCATGTGGTTTTAGGG	GTCACGTTTCGGGATGAAAG	5	TG	1
LKH-004_A15_F	GGCTGGAATAAGACGGTGAA	GAGCCGTGTCCATTGAGATT	6	CA	-
LKH-004_A16_R	AACCAGCTTCCTCAGGATCA	ACTGCTGGGGAAGGGATATT	5	TTA	1
LKH-004_C01_R	GCGCGTAATAACCATTTTCC	TCTGGCAAAAGAGAATCATCTAAA	5	GA	-
LKH-004_C03_R	AATTTTTGAATCCGCCTCTG	TCGTCAGGTGGTTTGTGAAA	5	ATG	4
LKH-004_C06_F	ATACCAGAGCGTTGGTCCTG	AGGGACCTTCGATAATGCTG	6	ATT	3
LKH-004_C13_F	TCTGCGCATCTCTGTTTTTG	TGTTTGAAGGGTGGGGATAA	5	AT	1
LKH-004_C18_F	CGCATCCTTTAGAGCAGCAT	GAAATGGACGTTAATCGCAGT	5	AG	-

LKH-004_D16_F	TACCGCAGCAATTCTCAGTG	TTTTTCTCGGAAGCTGAGGA	5	ATA	1
LKH-004_D21_R	TCGCAAGGGAACTGAAACTT	ATTGACTCCCGGTCAAAGTG	5	ATA	4
LKH-004_E02_R	CAGTTGAGTTGAATTAAAAGAAATGC	TTACCCCACCTTGGAATTGA	5	TAT	2
LKH-004_E04_F	CCCCAATTCGACGTATCTGA	TACGATTAGGGACCGGGACT	5	CAA	3
LKH-004_E09_R	TCAATCGTGGTAGCGCATTA	TTGTTTACATCTCCGCAGCTAA	6	ATT	4
LKH-004_E16_F	TTGCCATAGCAACAAGTTCG	TCAGTCGTGGAAGACATACTGA	5	AT	3
LKH-004_G05_R	TTTCCGCTTTCAAAGATGCT	GGTGCAAAATAACGGATGGA	5	AG	2
LKH-004_G16_F	AAAAAGGCAGCACCATCTTG	GTGCTCTACGCGTTCCATTC	5	TC	1
LKH-004_G18_F	TACAGTGGCTGAGGGAGGTC	TGTTTTAGCCCCAACTCCTG	5	TAA	1
LKH-004_H07_F	CCTAACCAGGAAACAACATCAA	AACGGAAACGCAACCAAATA	10	TTA	7
LKH-004_H07_R	GGAATTCGTCAATTACAACCGTA	GAGCCTTACTGGCACTCGTC	5	AT	2
LKH-004_H11_R	TCGATTCTCAGTTCCAAGCA	GCGTAATACTCGAACACTGTGG	6	TA	1
LKH-004_H15_F	AGCCACAACAGTACCTTGGAA	GGATGTTACGGTAGACCCACA	11	AAGA	3
LKH-004_I03_F	AGGCATGCAAGCTTTAATCA	ATCTGTCTTTGGCTCCAAGC	7	TA	5
LKH-004_I24_F	GCTTCGAAAACCAGCCTGTA	TCAAGTGCCTGGAATAGATGG	7	AAT	3
LKH-004_J15_R	CTTTCTGAAATGTCCGGTGA	TTGTCCTCCACGTGGTCATA	36	TA	6
LKH-004_J16_F	TGCAAGCTTTTCCTCCAACT	TCGCCATCTCTTCACACAAG	5	TG	1
LKH-004_K05_R	CATAGCACCCCCGAATTAAA	CGCATGACCAACAGATGAAG	6	TC	2
LKH-004_K12_R	CAACCGTCCTTCGTTCAAAT	TTGTTGGAAAGCGACTGAAA	5	AAT	1
LKH-004_K17_F	CCAACGCTTCGCAATAAAGT	GCGGAACGTTCAGTCAATTT	5	GCC	-
LKH-004_L10_F	GCTGGAAACCAGCGACTTAG	CCTCCCCTCACAACACTTTC	5	TG	3
LKH-004_L19_F	AACCGCTACTTCGACCTTCA	GTGGTCATTCGCAGTTTGTT	8	TG	4
LKH-004_N01_R	CACTCGTAATCACGTGGTCCT	TGAACAAAACGAATGGGTCAT	10	GA	5
LKH-004_N03_R	ATTGGGTGGGAAATGTATGC	GAGGGAATCCACTTTGAAAATC	7	AAT	4
LKH-004_N06_F	TCGCTGAAGTAGGACCTTCG	GGTGTCCGCATATCAGAAGTG	37	TA	5
LKH-004_N20_F	GCATGCAAGCTTCAATAGCA	GAGGAGAACAAGAGCGGATG	5	CCG	4
LKH-001_B21_F	ACCATCCGAGACGCTATGTG	CATTTGTCACCTCACGGAAA	9	GA	7
LKH-001_C11_R	ATCGCGTGAAAGTCCAAGAA	GAACATAGGCAACCCGACAT	10	AG	2
LKH-001_C14_R	TCTCCACGTGTCTCAGATCG	TTTGGTTGTGGAGAGTGGTG	5	TAA	2
LKH-001_D09_F	GCATGCAAGCTTCAATAGCA	GAATCCGCCTCTGCCTTAG	5	AAAT	1
LKH-001_E07_R	AGTCAGGCGACATCGTTACC	TCCTTAGAGGGCCAATCACA	6	TAA	7
LKH-001_E08_R	TGTACGCCTCGCATTAGTGA	TTCCCTTCGCATTCAAAGAC	7	TGTC	4
LKH-001_E10_F	GCTGAAACGTTTGCGTTGTA	CCCGTCATAAAATCCTTCCA	20	TA	-
LKH-001_E24_R	GGTCACGTGAACTGCTGATG	GTTCACTCCGATTCCACGAT	7	TAT	4
LKH-001_F13_F	GCAGGCATGCAAGCTTTATT	GCGGATTCAAAAATTTCAGC	6	AT	2
LKH-001_G02_R	ACACCCTGTCAAATGCAACA	GCCCGAAAAACTTTCCCTAT	5	CA	1

LKH-001_G09_R	CTGATTGACACAGCGGACAT	ATCCAAACGCAGTGGAAAAC	5	ATT	1
LKH-001_H02_R	TTTAGTAGGTGGGCCGGTTA	CGTGGCTCAGGTGGTAGTTT	5	TAG	1
LKH-001_H08_R	CTTCGTCACCTTGCTTCGTT	TAGAGCCATCAGCGCTAACA	12	CT	1
LKH-001_H09_F	GTGCGAAAACCGATACCATT	AACATCAACGCGAGGAAAAC	13	ATA	-
LKH-001_H12_F	TGCAAGCTTCTGCGTGATAG	GGAAAACGTCGAGAGACCAG	5	GA	1
LKH-001_I12_R	TTCCTTTAATGAGCTCAGACCA	TGTTAGAACACGATCTTACGTTGG	30	AT	-
LKH-001_I19_R	TTCGGGCTGTAGTTCGAGTT	GCATGCATCCATTCCTTTGT	5	CT	1
LKH-001_K08_R	TGTGACTCTCGCGGTAATTG	GAGGCCATCATCAACGCTAT	9	GT	1
LKH-001_K20_F	GGTCAGGTTTGGGAAGAACA	CACGTGGTCATACGCAGTTT	38	TA	3
LKH-001_L06_F	CCCTTAAAGCCCCACAAAA	TTTTTCCCTCCACGTGGTTA	10	AT	2
LKH-001_L18_R	TCTGCTGCAACAATAGGTGTG	GTCTGAGCTGCAAATGCTTG	6	AAT	3
LKH-001_N08_F	CGGTCGAAACATCATGAGC	AATTTCTGCGCGACACTTTT	5	TG	1
LKH-001_N12_F	CAGCATGCAAGCTTGAGAAA	GCGGTGGACGTACGTGTAAT	5	CTG	2
LKH-001_N16_R	GACTACCGGCATCACCCTTA	AAGGTAATTGTTTCTGTGGTTGG	6	AT	3
LKH-001_N24_F	ATGCAAGCTTTTTCGACCAC	GGGTGGAATTTCTCCCTTTC	5	AC	1
LKH-001_O10_F	GCGAGGACGAAAGTGGTAAC	AATCAGCACGCCCATTGTAT	37	TA	6
LKH-001_O10_R	AAGCAGCCATTTTCAATCGT	GTTTCGACCTCTTCGGATCA	6	GA	7
LKH-001_O18_F	CCCTCCATGTGACATTCCTC	CAGCGAGTTCCCCATAGAAG	5	TCT	1
LKH-001_O21_F	GCTTTACGTACCGGAAACGA	TCGAATTCTTTTGTAATCCAGACA	42	AT	2
LKH-001_P16_R	AATTCGTCGCGAGTTTTCAC	GCGGGACTTCTTTTTACGAA	5	GT	1
LKH-002_A03_F	CGAGAAATGGAATCGACGTT	CCCGTTCTTTCACCTACTGC	5	AT	1
LKH-002_A03_R	TTGCCTCATACGAGCACAGT	TGCAAATTATGCAACCCTCA	14	ATA	-
LKH-002_A16_F	CATGCAAGCTTGTCAAACA	TATTTTCCCCACGTGCATTT	18	TA	-
LKH-002_A19_R	GCTTCAGACTTTTCGTTTCCA	ACCAGACGCAATTCAAGGAA	6	AAT	2
LKH-002_B05_R	GCATATCCGCCATCTTGACT	TGCACGTTTTCTTTTTCACG	6	AC	2
LKH-002_B11_F	AGCTTCCGTGTGAGAGGAAA	CATGAGCAAATGCAAAGCAC	8	TA	2
LKH-004_N21_R	CCTAAAACCCCAAGGGAAAA	GGGACGAGGATGACAAGAAA	5	TTAT	6
LKH-004_O09_F	TGATGCATCCATTTGTTCGT	GCGCACACACGAGTTATTGT	5	AC	1
LKH-004_O22_R	GCTCCAATTTGACGGAAAAA	TCAAATCCAGGAAGAAGGAAAA	5	CT	1
LKH-004_P15_R	TCTTGGACGCCTCGATAGTC	AAGAGACCAAAGTGCGCAAG	5	CGC	3
